# SalmonAct deciphers transcription factor regulatory activity in *Salmonella* transcriptomics

**DOI:** 10.1128/msystems.01239-25

**Published:** 2026-03-30

**Authors:** Marton Olbei, Balazs Bohar, Robert A. Kingsley, Tamas Korcsmaros

**Affiliations:** 1 Division of Digestive Diseases, Department of Metabolism, Digestion and Reproduction, Imperial College London 216729, London, United Kingdom; 2 Earlham Institute 150238, Norwich, United Kingdom; 3 Quadram Institute Bioscience 7308, Norwich, United Kingdom; Det Kongelige Akademi-Konservering, Copenhagen, Denmark

**Keywords:** *Salmonella*, transcriptomics, transcription factors, regulatory network

## Abstract

Foodborne *Salmonella enterica* infection remains a major public health threat due to its prevalence in food, ease of transmission, and increasing antibiotic resistance. Consequently, understanding the molecular mechanisms underlying *Salmonella* pathogenesis is crucial for guiding novel diagnostic, preventative, and therapeutic approaches. High-throughput transcriptomic technologies are now often employed in *Salmonella* research to quantify how gene expression changes in response to various conditions or mutations. But due to the high dimensionality of this data, resulting from the comparison of the expression of thousands of genes from multiple strains or culture conditions with complex interactions, interpretation remains a demanding task. To address this issue, we present SalmonAct, a comprehensive signed and directed prior knowledge resource for inferring transcription factor activities in *Salmonella*. This new resource expands the toolkit available for *Salmonella* functional analysis. Built as an extension of the SalmoNet2 database, SalmonAct can be used to infer the activity of 191 transcription factors in 5,991 regulatory interactions based on publicly available interaction and regulatory knockout data. SalmonAct enhances the interpretation of highly dimensional transcriptomic data by identifying both highly influential and minimally active transcription factors that drive the observed expression state. SalmonAct aids in bridging the gap between model and non-model organisms' functional analysis, and together with the SalmoNet2 resource, can be used for further downstream data analyses.

**IMPORTANCE:**

While modern high-throughput transcriptomics technologies provide effective ways of studying *Salmonella enterica*, the complexity of these datasets makes interpretation challenging. SalmonAct provides a new computational resource to connect gene expression changes with the activity of transcription factors, key regulators of bacterial adaptation and virulence. SalmonAct can help researchers progress from the analysis of gene expression profiles towards a clearer understanding of the regulatory programs driving the behavior of the pathogen. By integrating prior knowledge of transcription factor-target gene relationships, SalmonAct contributes to uncovering novel mechanisms of bacterial pathogenesis and supports the development of new approaches to combat *Salmonella* infections.

## INTRODUCTION

*Salmonella enterica*, a globally significant pathogen, causes a diverse range of illnesses, from mild gastroenteritis to life-threatening systemic infections (1). It poses a significant public health challenge due to its widespread presence in the food supply, the ease with which it can spread, and the increasing prevalence of antibiotic-resistant strains (2, 3). Therefore, a comprehensive understanding of the molecular mechanisms underlying *Salmonella* pathogenesis is crucial for developing novel diagnostic, preventive, and therapeutic approaches (4). High-throughput transcriptomic technologies play a pivotal role in identifying these mechanisms, by revealing how gene expression changes in response to environments encountered by the pathogen during its lifecycle, enabling targeted studies of key regulators and pathways (5).

As the cost of data generation continues to decrease, the volume of publicly available transcriptomic data sets is rapidly growing, making it increasingly important to develop methods for extracting functional information from these results (6, 7). While analyzing RNA level changes across conditions can provide valuable insights into a system’s state, the high complexity of these data can make interpretation challenging. Leveraging prior knowledge networks (PKNs), which represent known, experimentally validated relationships within a system, reduces data complexity and enhances statistical power. By aggregating signals from known target genes, these methods estimate transcription factor (TF) influence more robustly than by examining the expression of singular genes (8). Although prior knowledge resources are increasingly available for well-characterized model organisms including *Escherichia coli*, they remain limited for other important pathogens such as *Salmonella enterica* (4).

One such application of PKNs is the inference of transcription factor (TF) activity. TF activity is the influence a TF exerts on the transcriptome, evaluated through the expression of the TF’s target genes. TFs can bind specific DNA sequences to either activate or repress transcription. Their overall activity, or influence, can be inferred by examining whether their known target genes are up- or downregulated, in line with the TF’s mode of regulation (inhibition or stimulation). By assessing whether sets of target genes are up- or downregulated in a given data set, we gain a more complete view of TF function, rather than relying solely on the TF’s own differential expression status. In other words, this approach aggregates signals from multiple genes to identify whether a TF’s regulatory influence is broadly active or inactive under the condition of interest, based on the behavior of the TF’s known target genes.

PKNs for non-model organisms such as *Salmonella* are rare and tend to focus on different molecular mechanisms. For example, the KEGG (9) and BioCyc (10) databases collate *Salmonella*-specific metabolic pathways, the STRING database (11) contains protein associations, but neither of them contains gene regulatory interactions. Specifically for gene regulatory networks, the CollecTF (12) and PRODORIC (13) databases contain signed, directed, experimentally validated *Salmonella* interactions; however, their coverage is limited. Other resources, such as RegPrecise (14), contain a more expansive, manually curated set of regulatory interactions, and databases such as SalComRegulon (15) collate the effect of regulatory knockouts on the *Salmonella* transcriptome. Previously, we have developed SalmoNet2 (16), a multi-layered *Salmonella* interaction resource that simultaneously integrates protein-protein, transcriptional regulatory, and metabolic interactions to provide an enhanced view of *Salmonella* biology. Although SalmoNet2 includes a comprehensive gene regulatory layer for 20 *Salmonella* strains, these gene regulatory interactions are not signed, and thus cannot be used for transcription factor activity inference.

Here, we introduce SalmonAct, a signed and directed prior knowledge resource specifically developed for *Salmonella* transcription factor activity inference. SalmonAct is designed to work alongside established methods for inferring transcription factor influences. SalmonAct provides *Salmonella*-specific regulatory interactions that serve as input for established statistical methods used for activity inference. By combining transcription factor activities with transcriptomics data, this integration enables researchers to gain a deeper understanding of the mechanisms controlling *Salmonella* biology.

## RESULTS

### A novel prior knowledge network for *Salmonella*

To collate the signed and directed interactions between transcription factors and their regulated target genes, we integrated a prior knowledge network (PKN) from multiple information sources. Data were imported from the CollecTF, PRODORIC, RegPrecise, and RegulonDB databases, and regulatory perturbation data from the SalComRegulon data set (12–15, 17). The resulting PKN contains 5,991 signed and directed regulatory interactions for 191 transcription factors. 44% of interactions are inhibitory, and 56% are stimulatory. For further details on PKN construction, please see the Materials and Methods section.

The PKN can be accessed directly from the SalmoNet2 website (http://salmonet.org/) and the community standard NDEx network repository (https://doi.org/10.18119/N9XK69). Interactions are provided in a format that is directly accessible by applicable methods, such as decoupleR. DecoupleR is an R package and Python library developed to extract biological activities from omics data, with a multitude of methods, including their consensus decisions (8).

### Inferring transcription factor activities using the SalmonAct network

**Fig 1:**
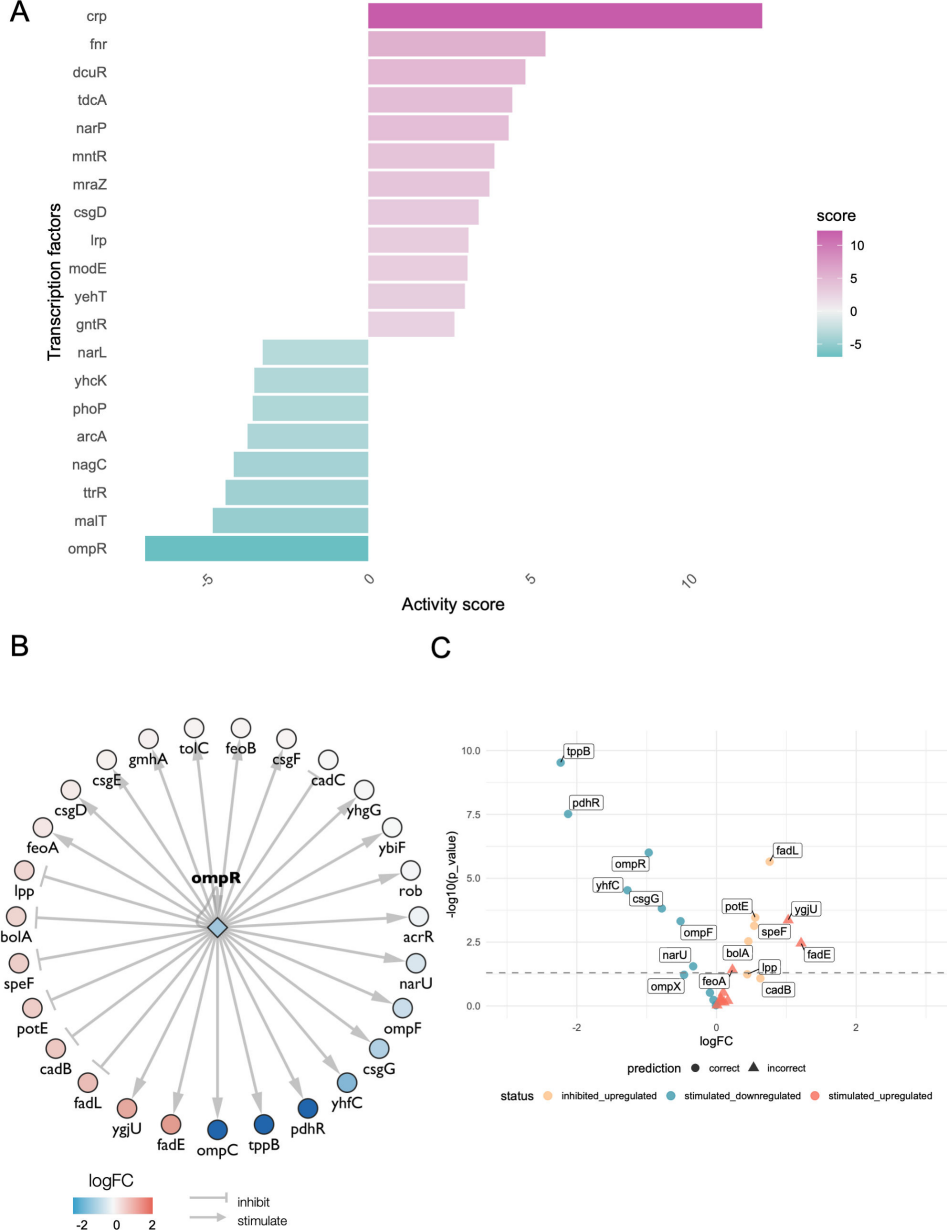
Transcription factor activity analysis on an ompR knockout data set. (**A**) Bar charts depicting the most and least active transcription factors in the ompR knockout vs. wild-type comparison. (**B**) OmpR targets and their differential expression levels, illustrating how the mode of regulation aligns with the observed behavior of its target genes. Because many genes normally activated by OmpR are downregulated and many that it represses are upregulated, its inferred activity is low. (**C**) Volcano plot depicting the differential expression status of OmpR targets, and whether their behavior was accurately predicted (shapes). The point color depicts the sign assigned to the target gene in the PKN and its differential expression status. We note that no target gene was inhibited and downregulated; hence, the category is missing from the plot.

To explore the potential of SalmonAct, we analyzed two *Salmonella enterica* transcriptomics data sets and determined the least and most active transcription factors in each case. In the first use case, we analyzed data for genes regulated by the OmpR regulon identified by culturing wild-type and *ompR* mutant *S*. Typhimurium strain SL1344 to mid-exponential phase (18). We determined the differentially expressed genes between the two groups (SL1344 ompR/SL1344 wildtype) and analyzed the results with SalmonAct and decoupleR. OmpR was found to be the least active transcription factor, as could be expected, following the knockout experiment (Fig. 1A). The master regulator *crp* was found to be the most active transcription factor. Crp fills a central role in the physiology of *Salmonella*, integrating environmental signals through intracellular cAMP levels, which it binds as a cofactor (19). Figure 1B shows the expression levels of the target genes of OmpR following the knockout, illustrating how they contribute to its low activity score. Since targets OmpR normally activates are downregulated, and many of the targets it normally suppresses are upregulated, in the absence of OmpR, the TF achieves a low score and is therefore considered inactive. While the majority (19 out of 29) of OmpR target genes behave as predicted by the PKN and activity inference, a small number of edges are discordant with the expected direction of regulation. For example, *ygjU* and *fadE* are upregulated following OmpR knockout despite being annotated as stimulated by OmpR in the PKN (Fig. 1C). Quantifying the precision of the OmpR targets in SalmonAct, the regulon achieved a precision of 0.8 with significantly DE target genes (12 out of 15), or 0.65 when using all targets (19 out of 29).

**Fig 2:**
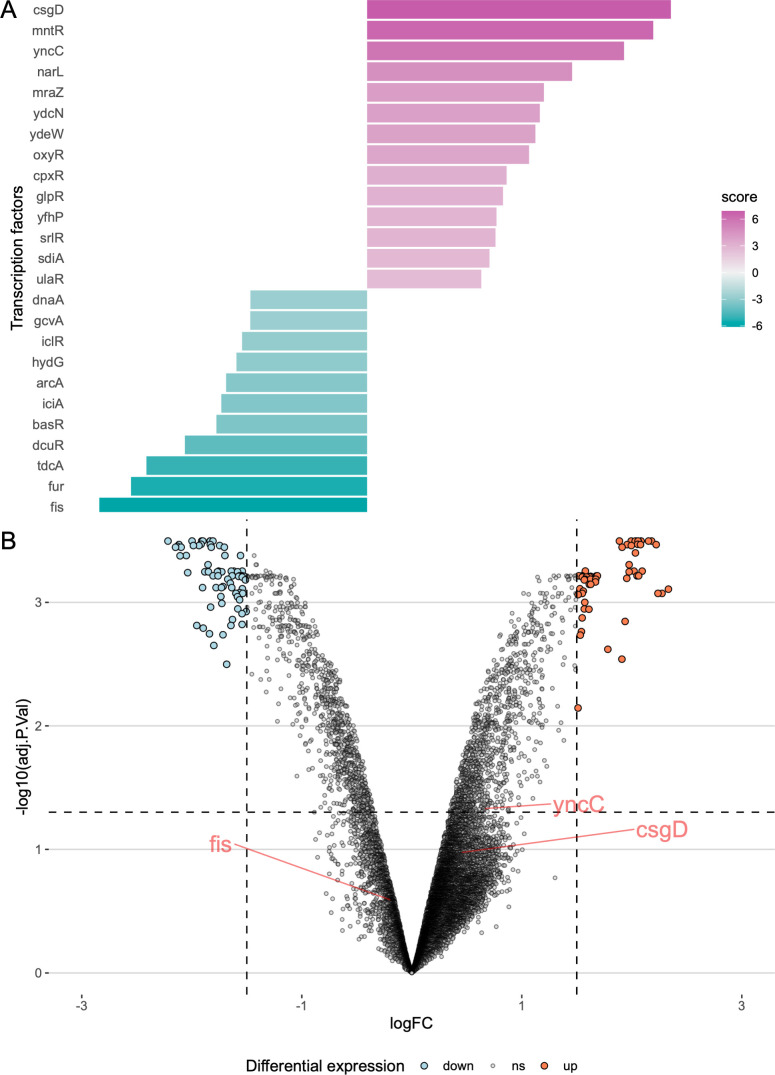
Transcription factor activity of *Salmonella* entering the stationary phase. (**A**) Top-ranked transcription factor activities based on differentially expressed genes between the stationary and log phase cultures. (**B**) The most active and inactive transcription factors, CsgD, YncC, and fis, are labeled. Although these TFs are not differentially expressed themselves, the collective behavior of their target genes suggests activation (or inactivation).

In the second set of analyzed transcriptomics data, we analyzed the differential expression of genes during log phase and stationary phase, in a culture of *S*. Typhimurium strain 14028S (20). Figure 2A shows the ranking of transcription factor activities. The highest-scoring transcription factor, CsgD, is the master regulator of biofilm development in *Salmonella*. The high activity is to be expected, as cells transition from motile cells to the stationary phase and begin biofilm formation (21). YncC (also known as McbR), the third-most active transcription factor, similarly stimulates biofilm formation in *E. coli* (22). The least active transcription factor, fis, fills a central role in regulating metabolic and type III secretion factors, targeting many of the SPI-1 and SPI-2 genes (23, 24). Figure 2B shows the differential expression results on a volcano plot, with YncC, CsgD, and fis highlighted. Neither transcription factor is differentially expressed, but by involving data from the collective behavior of their target genes, we can get a clearer idea of how the system as a whole is behaving instead of having to rely on the differential expression status of individual transcription factors. Figure S1 shows the differential expression status of the target genes of the highlighted transcription factors.

## DISCUSSION

In this work, we have created a novel resource to infer transcription factor activity in *Salmonella*. The goal of this extension to SalmoNet2 is to bring more functional analysis tools available for the study of model organisms to the *Salmonella* community. Combining transcription factor activity inference with other recently introduced functional analysis tools to the *Salmonella* field, such as pathway enrichment (25), has the potential to assist in the characterization and a greater understanding of transcriptomics experiments. Utilizing transcription factor activities could aid antibiotic exposure profiling by inferring TF activity shifts following antibiotic stresses; better understanding of pathogen responses to host environmental stresses, such as oxidative or bile stress; while dual-RNA-seq studies could highlight how *Salmonella* TF activities correlate with host pathways.

By determining the activity levels of transcription factors from transcriptomics data, we have a chance to interpret the system in a way that both reduces the dimensionality of transcriptomics data and increases its robustness, as the activity measure of the individual transcription factors gets determined by the collective behavior of their target genes (26) rather than single data points. To help interoperability with existing tools, we created SalmonAct in the format specifically required for decoupleR, a tool for activity estimation from ‘omics data. Naturally, the resource can also be utilized with other applicable standalone activity inference approaches, such as VIPER (27). We also provide helper scripts and tutorials on the SalmoNet2 website to help interested parties analyze their data with both sets of tools.

An important consideration when interpreting TF activity scores is that transcription factor activity *in vivo* is determined by multiple factors beyond gene expression levels. In both *E. coli* and Salmonella, TFs often require cofactors, post-translational modifications such as phosphorylation, or interactions with other regulatory proteins to exert their regulatory effects. This means that many TFs function is capable of both activating and repressing the same set of target genes depending on specific conditions. SalmonAct’s regulatory sign assignments represent snapshots of TF-target relationships under particular experimental contexts, sourced from different data sources.

As such, the predictive power of the method is determined by the quality and coverage of the underlying PKN. Our novel PKN consists of interactions from available regulatory databases, interolog transfer from *E. coli,* and an extensive source of regulatory perturbation experiments from SalComRegulon. Regulogs may be appropriate for *Salmonella* strains with the greatest synteny to *E. coli*, but may also be a limiting factor in the case of strains that have undergone extensive pseudogenization in many of their genes, as is the case for many extraintestinal *Salmonella* strains as they adapted to an invasive lifestyle (3). The predictive power of SalmonAct can nonetheless be improved in the future as more relevant data sets become available that elucidate the behavior and targets of individual transcription factors in *Salmonella,* and incorporate more condition and cofactor specific regulatory information.

## MATERIALS AND METHODS

### Establishing the SalmonAct PKN

To establish the PKN, we combined signed, directed regulatory interactions from a number of sources:

- PRODORIC and CollecTF: experimentally validated, *Salmonella*-specific signed and directed transcription factor-target gene interactions were included from the PRODORIC and CollecTF databases (12, 13).
- SalComRegulon (15): *Salmonella*-specific interactions were inferred from the SalcomRegulon data set, a transcriptomics compendium containing 18 transcription factor knockouts of *S*. Typhimurium 4/74. The perturbation data deposited here were used to determine the inhibitory or stimulatory nature of the transcription factor knockouts and their regulated target genes. A |log2FC| ≥ 3 cutoff of the responding genes was used as a threshold for inclusion. As the SalcomRegulon data contains regulatory knockouts, we determined their mode of regulation based on the direction of change following the knockout, that is, log2FC ≥ 3 was encoded as inhibitory (−1), while log2FC ≤ −3 wass considered activatory (+1).
- RegulonDB: information was retrieved from RegulonDB (28), a database of transcriptional regulation in *Escherichia coli* K-12. These conserved interactions were imported based on the concept of regulogs (29), using the protein orthology relationships established for SalmoNet2 (16).
- RegPrecise: manually curated, signed TF-target gene relationships were downloaded from the RegPrecise website (14). Interactions with no annotated mode of regulation were removed.

We then harmonized the confidence levels from the resources (Confirmed, Strong, and Weak) ensuring consistent annotation across the network, to help users fine-tune their search terms. Confirmed *Salmonella*-specific interactions had robust experimental validation from CollecTF or PRODORIC. RegulonDB regulon interactions were allocated into Strong and Weak categories, based on the quality of *E. coli* interactions, as established by RegulonDB initially. Most RegPrecise interactions were noted as “Strong”; however, for TFs with an ambiguous mode of regulation, the primary annotated mode was used, and these associations were categorized as “Weak” due to this ambivalence. Interactions mapped from SalComRegulon perturbation data were assigned into the “Weak” category, as the perturbations do not capture the differences between primary or secondary order regulatory effects caused by the transcription factor hierarchy present in the cell; these edges are sign-consistent hypotheses, not direct binding evidence. Interactions present in multiple resources were collapsed, and the sources were listed in the corresponding column. In the case of partially matching interaction data, the higher quality interactions were kept. In cases where the partially matching interactions were in the same category (Weak), the more *Salmonella*-specific data sources were kept (RegPrecise and SalComRegulon > RegulonDB). From the initially assembled PKN, orthology-based mapping was done for all strains included in SalmoNet2, using the orthologous relationships established with OMA (30) for SalmoNet2. We provide a mapping table on the project GitHub page to enable availability for all 20 strains in the database. The target genes in the mapped resources were noted using strain-specific locus tags.

### Inferring transcription factor activity of *Salmonella* regulons

Differential expression for the selected transcriptomics data sets (GSE35938 and GSE11486) was established using GEO2R with default settings. In the GSE35938 data set wild-type strains grown in LB were contrasted to *ompR* mutants, while in the GSE11486 data set, wild-type strains grown in log phase were compared to wild-type strains grown in stationary phase.

The R package decoupleR (version 2.10) was used to infer the activities from the PKN (“Strong” and “Confirmed” interactions) and transcriptomics data sets. The univariate linear model (ULM) approach was used to determine transcription factor activity, with the minimum size of target sets set to 5, and a significance cutoff of 0.05.

The ULM approach creates a linear model where the predictor variable is the interaction weight between the transcription factor (TF) and each gene (−1 or +1 based on the mode of regulation included from the sources the network was built from), and the response variable is the expression level of the genes. This way, ULM evaluates how well the interaction weights of a TF predict the expression of its target genes, by fitting a linear regression resulting in a slope value that represents the relationship between the TF’s interaction weights and gene expression. A positive slope means that as the interaction weight increases, the gene expression also tends to increase, indicating the TF is active. Conversely, a negative slope would suggest that the TF is inactive. The score for each TF’s activity is derived from the *t* value of the slope, which indicates the strength and significance of the relationship. A high positive *t* value means strong activation of the TF, while a high negative *t* value indicates inactivity. While the ULM method was used in these examples, decoupleR can perform other approaches aimed at TF activity estimation with the same input data, such as VIPER or AUCell (27, 31), and can provide a consensus score as well utilizing all 11 methods (returning a mean *z*-score across methods). Importantly, ULM scores each TF independently. When a target gene is regulated by multiple TFs, it contributes to the activity score of each regulator independently. Other methods such as VIPER can incorporate a pleiotropy correction (also referred to as “shadow analysis”) into the analysis, which penalizes regulators when there is a target overlap between different regulators. Users should consider which method is most appropriate for their biological question, particularly in cases where combinatorial regulation is expected to play a significant role.

Precision of the OmpR regulon was calculated by assessing the concordance between the predicted mode of regulation and observed behavior for every differentially expressed OmpR target gene (*P* ≤ 0.05), by dividing the number of true positives by all observations.

*P* values were adjusted using the “fdr” method using the p.adjust function in R. R scripts to replicate the differential expression results and transcription factor activities can be found in the project github repository: https://github.com/korcsmarosgroup/salmonella-TF-activity.

## Data Availability

The SalmonAct PKN is available on the project GitHub repository (https://github.com/korcsmarosgroup/salmonella-TF-activity), the SalmoNet2 website (http://salmonet.org), and the NDex database (https://doi.org/10.18119/N9XK69).

## SUPPLEMENTAL MATERIAL

Supplemental material msystems.01239-25-s0001.docx Table S1 and Fig. S1.

ASM does not own the copyrights to Supplemental Material that may be linked to, or accessed through, an article. The authors have granted ASM a non-exclusive, world-wide license to publish the Supplemental Material files. Please contact the corresponding author directly for reuse.
