# Supplementary material for: SalmonAct deciphers transcription factor regulatory activity in Salmonella transcriptomics
Source: mSystems. 2026 Mar 30;11(4):e01239-25. doi: 10.1128/msystems.01239-25 (PMC13098278; doi:10.1128/msystems.01239-25)
Supplement: Supplemental material — Table S1 and Fig. S1. [file msystems.01239-25-s0001.docx]

Supplemental File

Table S1: List of resources, transcription factors and source serovars or strains used to construct the PKN

| **Resource** | **Number of TFs** | **List of regulators** | **Serovars or strains** | **Address** |
| --- | --- | --- | --- | --- |
| PRODORIC | 16 | ArcA, CpxR, CsgD, Fis, Fur, H-NS, HilC, HilD, OmpR, PhoP, RamA, SlyA, CysB, FruR, MetR, Lrp | Salmonella enterica, S. Typhimirum str. 14028S | <https://www.prodoric.de/matrix/?term=Salmonella> |
| CollecTF | 10 | AraC, CRP, CueR, Fur, LeuO, OmpR, Phd, PhoP, PmrA, RpoN | S. Typhimirum str. 14028S, S. Typhimirum str. LT2, S. Typhi str. Ty2 | <http://www.collectf.org/browse/view_motif_reports_by_taxonomy/26/> |
| SalComRegulon | 18 | rpoE, fur, hilD, slyA, phoB/R, fliZ, ompR/envZ, ssrA/B, ssrA, ssrB, phoP/Q, phoP, barA/sirA, rpoS, hfq, hilA, hilC, hilE | S. Typhimirum str. 4/74 | Colgan et al., 2016 |
| RegulonDB_strong | 136 | fis, rob, tyrR, iciA, cpxR, modE, fnr, crp, mlc, cadC, tcuR, hns, soxS, oxyR, phoB, cysB, rcsB, lrhA, arcA, yijC, nhaR, lrp, fhlA, pspF, treR, marA, yfhP, phoP, narL, narP, ssrB, emrR, argR, fadR, sdiA, iclR, dnaA, nagC, norR, zur, putA, celD, ygaE, gntR, uxuR, yjfQ, trpR, malT, pdhR, yifA, slyA, ygiX, araC, rhaS, melR, cytR, gcvA, torR, gatR, yhcK, ada, birA, deoR, galS, galR, lexA, metR, purR, rhaR, ilvY, cspA, fruR, leuO, marR, nadR, rbsR, soxR, tdcA, csgD, xylR, caiF, lctR, hydG, adiY, kdgR, allR, ydeW, baeR, acrR, nsrR, pepA, bolA, alaS, yabN, yohL, nrdR, rstA, ycdC, citB, stpA, creB, acrS, ydhM, yncC, yfhA, yqhC, mlrA, aidB, ycfQ, fliZ, yehT, basR, ydfH, ykgD, mraZ, ydcN, copR, yeaM, yjjQ, sirA, ybiH, yhaJ, glnG, ompR, dgoR, yihW, fur, dcuR, kdpE, uhpA, cueR, nikR, zntR, mntR, metJ, rpoN | Escherichia coli mapped to S. Typhimirum str. SL1344 through protein orthology | <https://regulondb.ccg.unam.mx/> |
| RegulonDB_strong | 90 | fis, rob, tyrR, iciA, cpxR, modE, fnr, crp, mlc, cadC, tcuR, hns, soxS, oxyR, phoB, cysB, rcsB, arcA, lrp, marA, yfhP, rtcR, phoP, narL, narP, ssrB, emrR, fadR, dnaA, nagC, cdaR, zur, gntR, idnR, uxuR, malT, pdhR, slyA, ygiX, araC, cytR, torR, deoR, galS, galR, lexA, metR, purR, asnCb, cspA, dsdC, fruR, fucR, gutM, srlR, leuO, lysR, nadR, csgD, xapR, hydG, prpR, kdgR, ybbS, baeR, acrR, nsrR, bolA, rstA, ycdC, stpA, creB, fliZ, copR, yjjQ, yhaJ, glnG, ompR, fur, accB, dcuR, cueR, ydcI, mntR, metJ, yhcS, yfeC, yidZ, yciT, rpoN | Escherichia coli mapped to S. Typhimirum str. SL1344 through protein orthology | <https://regulondb.ccg.unam.mx/> |
| RegPrecise | 71 | AcrR, AllR, AraC, ArgR, BirA, Crp, CueR, DeoR, DgoR, FabR, FadR, Fnr, FruR, FucR, Fur, GalR/GalS, GcvA, GlpR, GntR, HexR, HutC, IclR, IdnR, IlvY, IscR, KdgR, LeuO, LexA, LldR, LsrR, MarR, MetJ, MetR, MntR, ModE, MprA, NadR, NagC, NanR, NarP, NhaR, NikR, NorR, NrdR, NsrR, NtrC, PdhR, PhnR, PurR, QorR, RbsR, RcnR, RhaR, RhaS, RspR, RutR, SdaR, SgaR, SiaR, SoxR, SrlR, SL1555, TalR, TrpR, TtrR, TyrR, UlaR, UxuR, YiaJ, ZntR, Zur | S. Typhimirum str. LT2 | <https://regprecise.lbl.gov/genome.jsp?genome_id=354> |


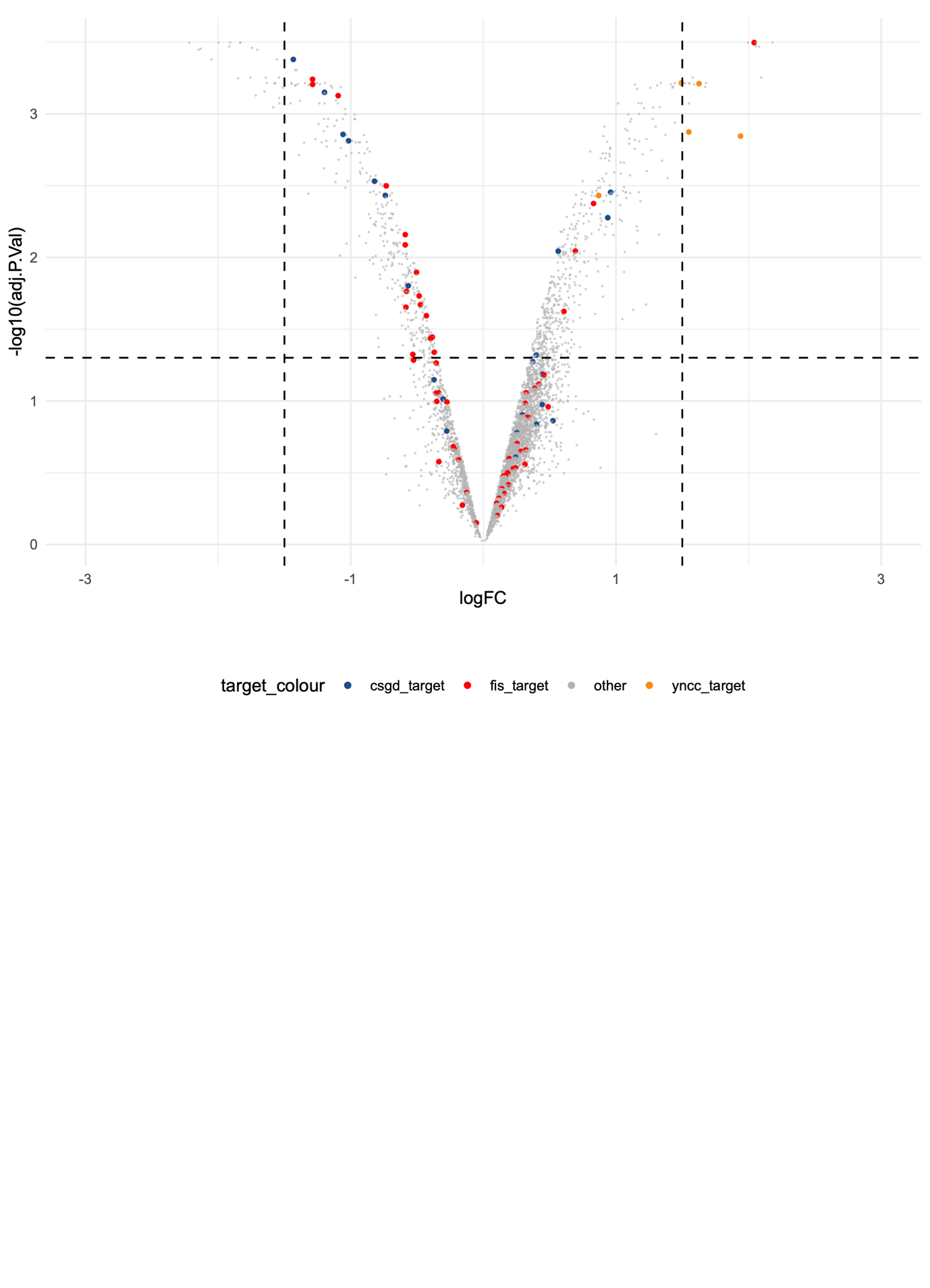


Figure S1: Differential expression status of the fis, csgD and yncC target genes in the log vs stationary phase experiment.
